# Patients with allergic asthma have lower risk of severe COVID-19 outcomes than patients with nonallergic asthma

**DOI:** 10.1186/s12890-022-02230-5

**Published:** 2022-11-14

**Authors:** Thomas R. Murphy, William Busse, Cecile T. J. Holweg, Yamina Rajput, Karina Raimundo, Craig S. Meyer, Arpamas Seetasith, Sachin Gupta, Ahmar Iqbal, Robert J. Kaner

**Affiliations:** 1 ENT and Allergy Partners of Charleston, Charleston, SC USA; 2 University of Wisconsin School of Medicine and Public Health, Madison, WI USA; 3 Genentech, Inc., South San Francisco, CA USA; 4 Weill Cornell Medicine, New York, NY USA

**Keywords:** Allergic asthma, Allergy, Asthma, Atopic, Coronavirus, COVID-19, Nonallergic asthma, Pandemic, SARS-CoV-2, Respiratory

## Abstract

**Background:**

Although asthma does not appear to be a risk factor for severe coronavirus disease 2019 (COVID-19), outcomes could vary for patients with different asthma subtypes. The objective of this analysis was to compare COVID-19 outcomes in real-world cohorts in the United States among patients with asthma, with or without evidence of allergy.

**Methods:**

In a retrospective analysis of the COVID-19 Optum electronic health record dataset (February 20, 2020–January 28, 2021), patients diagnosed with COVID-19 with a history of moderate-to-severe asthma were divided into 2 cohorts: those with evidence of allergic asthma and those without (nonallergic asthma). After 1:1 propensity score matching, in which covariates were balanced and potential bias was removed, COVID-19 outcomes were compared between cohorts.

**Results:**

From a COVID-19 population of 591,198 patients, 1595 patients with allergic asthma and 8204 patients with nonallergic asthma were identified. After propensity score matching (*n* = 1578 per cohort), risk of death from any cause after COVID-19 diagnosis was significantly lower for patients with allergic vs nonallergic asthma (hazard ratio, 0.48; 95% CI 0.28–0.83; *P* = 0.0087), and a smaller proportion of patients with allergic vs nonallergic asthma was hospitalized within − 7 to + 30 days of COVID-19 diagnosis (13.8% [*n* = 217] vs 18.3% [*n* = 289]; *P* = 0.0005). Among hospitalized patients, there were no significant differences between patients with allergic or nonallergic asthma in need for intensive care unit admission, respiratory support, or COVID-19 treatment.

**Conclusions:**

Asthma subtype may influence outcomes after COVID-19; patients with allergic asthma are at lower risk for hospitalization/death than those with nonallergic asthma.

## Background

Coronavirus disease 2019 (COVID-19), caused by severe acute respiratory syndrome coronavirus 2 (SARS-CoV-2), can result in life-threatening respiratory symptoms [1, 2]. Pre-existing chronic respiratory diseases, such as chronic obstructive pulmonary disease or interstitial lung disease [3–6], have been shown to be associated with poorer outcomes after SARS-CoV-2 infection, but evidence that asthma is also a risk factor is less clear. Studies investigating the relationship between asthma and COVID-19 have reported variable findings, although the general consensus is that asthma is not associated with a greater risk of severe outcomes such as hospitalization [3, 6–12], although this may be complicated by the use of biologics [13].

Interestingly, studies have reported that COVID-19 outcomes may be better for patients with allergic asthma than for patients with nonallergic asthma. For example, analysis of UK Biobank data found that nonallergic asthma was significantly associated with severe COVID-19, whereas allergic asthma had no association with severe COVID-19 [8]. In addition, a South Korean nationwide cohort analysis found that patients with nonallergic asthma had a greater risk of SARS-CoV-2 infection and poorer clinical outcomes than patients with allergic asthma [14]. Similarly, a recent cohort study in the United States found that, compared with patients with nonallergic asthma, patients with allergic asthma were half as likely to be hospitalized with COVID-19 [15].

Because outcomes associated with COVID-19 may differ by data source (e.g., insurance database vs hospital records), and by country (e.g., patients’ demographic characteristics, comorbidities, and background asthma medications), we conducted a real-world retrospective electronic health record (EHR) database analysis in the United States. To assess potential differences between asthma subtypes, COVID-19 outcomes, including deaths, hospitalizations, and interventions and treatments, were evaluated and compared in patients with and without evidence of allergic asthma.

## Methods

### Database

This was a retrospective analysis of data from the COVID-19 Optum EHR database (Optum Inc., Eden Prairie, MN, USA), which includes medical records sourced from high proportions of ambulatory, hospital, and integrated delivery networks throughout the United States, regardless of insurance provider. Data for patients of any age (capped at birth year of 1930 and earlier) with confirmed COVID-19 between February 20, 2020 and January 28, 2021, were used.

The index date for COVID-19 diagnosis was the earliest date of presumed diagnosis or laboratory-confirmed SARS-CoV-2 infection, defined according to the US Centers for Disease Control and Prevention (CDC) guidelines, which include an *International Classification of Diseases, Tenth Revision, Clinical Modification* (ICD-10-CM) diagnosis code of U07.1 (COVID-19, virus identified) or U07.2 (COVID-19, virus not identified) [2]; a positive polymerase chain reaction diagnostic test for SARS-CoV-2; or an ICD-10 diagnosis code of B97.29 (other coronavirus as the cause of diseases classified elsewhere) without a negative diagnostic test within a 14-day window (± 7 days) [16].

To protect patient anonymity and confidentiality in compliance with the Health Insurance Portability and Accountability Act of 1996, all data used in this analysis were deidentified. The study was conducted according to the International Conference on Harmonisation E6 Guidelines for Good Clinical Practice and the principles of the Declaration of Helsinki 2013. The study did not require institutional review board approval because only deidentified data were included. Administrative permissions were not required to access the data.

### Cohort identification

Patient records were included in the analysis if they had evidence of persistent moderate-to-severe asthma, defined as ICD-10-CM J45.4X or J45.5X [17], at any time since October 2015. The cohort of patients with asthma with evidence of allergy (referred to as allergic asthma) was defined by a positive specific immunoglobulin E (IgE) serum test (IgE ≥ 0.35 kU/L), or skin prick test (Current Procedural Terminology code 95004) ordered by a relevant specialist (e.g., allergist, pulmonologist, dermatologist), or by a record of omalizumab use (Xolair®, Genentech, Inc., and Novartis Pharmaceuticals Corporation; Healthcare Common Procedure Coding System J code J2357). The cohort of patients without evidence of allergic asthma (referred to as nonallergic asthma) was defined as failing to meet the criteria for allergic asthma plus absence of any allergic comorbidities within 360 days before the index date. Patient demographics, clinical characteristics, and asthma medication use in the 12 months before the COVID-19 index date were extracted from the database. Patients were followed up from the index date through the end of data follow-up (date of the last record) or death, whichever occurred first.

### Propensity score matching

After identification of cohorts, 1:1 propensity score matching was undertaken to obtain 2 comparable populations for evaluation in which specified key demographic and clinical covariates were balanced and potential bias in between-cohort comparisons from measured confounding factors was removed [18]. The propensity score was defined as the probability of being in the allergic asthma cohort vs being in the nonallergic asthma cohort, conditional on the demographic and clinical variables in a logistic regression model. The variables used for matching included demographic characteristics (age [mean and category], sex, race, ethnicity, geographical region, index month [to account for temporal changes in diagnosis and management of COVID-19]), and asthma status (severity and medication categories [excluding biologics]). Clinical risk factors for severe COVID-19 illness were selected according to assignment of “strong and most consistent” or “mixed” evidence levels assigned based on published literature, CDC evidence, and practicing medical doctors (authors). Charlson Comorbidity Index and CDC definitions [19] were aligned to give a consolidated list of clinical variables, and ICD-10-CM codes assigned to these to facilitate identification in the EHR database. These included cancer, cardiovascular disease, cerebrovascular disease, chronic pulmonary disease, hypertension, obesity, pregnancy, smoking status, and type 2 diabetes. Patients with allergic asthma were matched 1:1 to patients with nonallergic asthma on the propensity score using greedy nearest neighbor algorithm with a caliper width of 0.1.

### Outcomes

The outcomes evaluated included numbers of patient deaths from any cause after COVID-19 index date; number of deaths within 30 days after COVID-19 index date; number of deaths from any cause after COVID-19 index date among hospitalized patients; hospitalizations and emergency department (ED) visits from 7 days before to 30 days after COVID-19 index date; intensive care unit (ICU) admission; length of stay (days); interventions, such as invasive or noninvasive ventilation; and treatments for COVID-19. Health care resource use was assessed as hospital resource use (hospitalizations and ED visits) and outpatient visits (visits to a health care practitioner office, nonspecified, COVID-19–related, or asthma-related). Selection of the COVID-19 treatments evaluated was based on medical opinion and published treatments (to date of analysis). COVID-19 outcomes not requiring hospitalization, such as changes in senses of smell and taste, outpatient visits (all-cause, asthma-related, COVID-19–related visits to a health care practitioner office), and hospital resource use rates (as resource rate ratios) were also evaluated.

### Statistical analysis

Descriptive analyses stratified by allergic asthma vs nonallergic asthma were performed for continuous variables using means, medians, and SDs, and for categorical variables using counts and percentages. Unadjusted differences in baseline characteristics between the 2 asthma cohorts were tested using nonparametric Mann–Whitney *U* tests for continuous variables, and χ^2^ tests and Fisher’s exact texts (with small cell counts) for categorical variables [20]. Poisson regression models were used to estimate hospital and outpatient care resource use counts per 1000 days and rate ratios, with 95% CIs and χ^2^ testing of regression coefficients [21]. The relative hazard of death comparing asthma groups and hazard ratios with 95% CIs was estimated using a Cox proportional hazards model. All analyses were performed using SAS Studio Release 3.7 (Enterprise Edition; ©2012–2017; SAS Institute Inc., Cary, NC, USA). Statistical significance threshold was set a priori using 2-sided tests at *P* < 0.05.

## Results

### Population characteristics

**Fig. 1:**
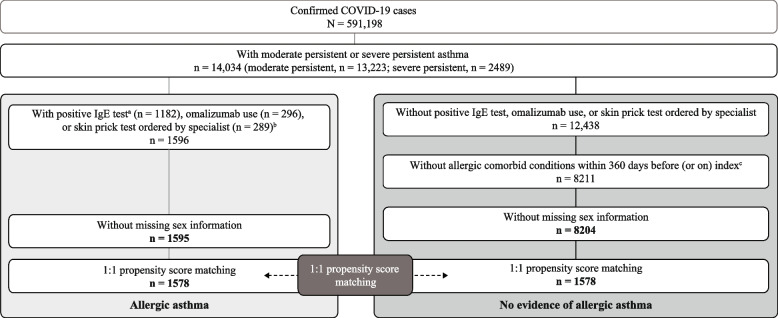
Study flow chart. ^a^ Cutoff value for positive immunoglobulin E (IgE) test was 0.35 kU/L or greater. ^b^ Not mutually exclusive. ^c^ Allergic comorbid conditions include allergic rhinitis, acute sinusitis, chronic sinusitis, anaphylaxis, conjunctivitis, dermatitis, food allergy, and urticaria. *COVID-19* coronavirus disease 2019

A total of 591,198 records were identified for patients who had been diagnosed with COVID-19 during the study period. Among patients with an asthma diagnosis, considerably fewer patients (*n* = 1595 [0.3%]) had a diagnosis of allergic asthma than nonallergic asthma (*n* = 8204 [1.4%]; Fig. 1). Patients with allergic asthma were slightly younger (mean [SD] age, 46.1 [18.8] years vs 49.4 [19.7] years; *P* < 0.0001) and more likely to have severe asthma (26.5% [*n* = 422/1595] vs 6.5% [*n* = 533/8204]).

Propensity score matching yielded study cohorts for allergic and nonallergic asthma that both included 1578 patients (Table 1). Patients with allergic or nonallergic asthma were broadly similar in terms of clinical characteristics and clinical risk factors for severe COVID-19, and there were more females than males in both cohorts. There were differences before COVID-19 diagnosis in asthma medication (such as use of biologics) due to differences in the treatment of allergic and nonallergic asthma. A higher proportion of patients with allergic asthma than nonallergic asthma had a history of severe asthma within the previous 360 days (25.8% [*n* = 407] vs 20.3% [*n* = 321]; *P* < 0.0001), although the overall Charlson Comorbidity Index scores were similar for the matched cohorts.

**Table 1:** Demographic and clinical characteristics of the propensity score–matched study cohorts

Baseline measure, n (%) unless stated	Nonallergic asthma (*n* = 1578)	Allergic asthma (*n* = 1578)	*P* value
Age (years), mean (SD)	45.8 (19.7)	46.2 (18.9)	0.4366
Female	1067 (67.6)	1061 (67.2)	0.8197
Race			0.9111
Black	352 (22.3)	353 (22.4)	
Asian	36 (2.3)	35 (2.2)	
White	948 (60.1)	962 (61.0)	
Other/unknown	242 (15.3)	228 (14.4)	
Ethnicity			0.6903
Hispanic	235 (14.9)	221 (14.0)	
Not Hispanic	1219 (77.3)	1239 (78.5)	
Unknown	124 (7.9)	118 (7.5)	
Region			0.6631
Midwest	726 (46.0)	759 (48.1)	
Northeast	563 (35.7)	523 (33.1)	
Other/unknown	39 (2.5)	38 (2.4)	
South	170 (10.8)	173 (11.0)	
West	80 (5.1)	85 (5.4)	
Asthma severity (within 360 days before or on index)			0.0001
Moderate	610 (38.7)	620 (39.3)	
Severe	321 (20.3)	407 (25.8)	
No moderate or severe asthma^a^	647 (41.0)	551 (34.9)	
Absolute blood eosinophils (× 10^3^/µL), mean (SD)	0.17 (0.24)	0.19 (0.22)	0.0001
Smoking status			0.892
Current smoker	92 (5.8)	96 (6.1)	
Never smoked	941 (59.6)	945 (59.9)	
Previously smoked	422 (26.7)	425 (26.9)	
Unknown	123 (7.8)	112 (7.1)	
Charlson Comorbidity Index score, mean (SD)	2.8 (2.9)	2.8 (2.9)	0.5528
Comorbidities and clinical risk factors for severe COVID-19 illness			
Hypertension	634 (40.2)	642 (40.7)	0.7717
Obesity (body mass index ≥ 30 kg/m^2^)	486 (30.8)	490 (31.1)	0.8776
Type 2 diabetes mellitus	316 (20.0)	328 (20.8)	0.5961
Cardiovascular disease	241 (15.3)	227 (14.4)	0.4832
Cerebrovascular disease	63 (4.0)	59 (3.7)	0.7119
Chronic kidney disease	112 (7.1)	94 (6.0)	0.1946
Chronic obstructive pulmonary disease	361 (22.9)	373 (23.6)	0.6131
Interstitial lung disease	126 (8.0)	152 (9.6)	0.1025
Primary cancer	64 (4.1)	63 (4.0)	0.9278
Metastatic cancer	9 (0.6)	9 (0.6)	1.0000
Immunocompromised	48 (3.0)	46 (2.9)	0.8341
Pregnancy	40 (2.5)	36 (2.3)	0.6423
Sickle cell disease	10 (0.6)	10 (0.6)	1.0000
Baseline medication use			
ICS, ICS-LABA, or LABA without LTRA	467 (29.6)	446 (28.3)	0.4097
ICS, ICS-LABA, or LABA plus LTRA	723 (45.8)	750 (47.5)	0.3354
Any oral corticosteroids	896 (56.8)	924 (58.6)	0.3131
Theophylline	12 (0.8)	23 (1.5)	0.0615
Tiotropium	11 (0.7)	25 (1.6)	0.0189
Any biologic	45 (2.9)	250 (15.8)	< 0.0001

*COVID-19* coronavirus disease 2019, *ICS* inhaled corticosteroids, *LABA* long-acting β-agonist, *LTRA* leukotriene receptor agonist

^a^ Not seen in the last year

### Deaths

More patients with nonallergic asthma than allergic asthma died (from any cause) after a diagnosis of COVID-19 (2.4% [*n* = 38] vs 1.2% [*n* = 19]; *P* = 0.0111; Table 2). Similarly, death from any cause within 30 days of COVID-19 diagnosis also occurred more frequently in patients with nonallergic asthma than allergic asthma (2.0% [*n* = 32] vs 0.9% [*n* = 14]; *P* = 0.0266). Among patients who visited the ED or who were hospitalized between 7 days before and 30 days after the index date, a higher proportion of patients with nonallergic asthma than allergic asthma died (from any cause).

**Table 2:** ED visits, hospitalizations, and deaths after COVID-19 diagnosis (propensity score–matched study cohorts)

Outcome, n (%)^a^	Nonallergic asthma (*n* = 1578)	Allergic asthma (*n* = 1578)	*P* value
Death from any cause	38 (2.4)	19 (1.2)	0.0111
Death from any cause within 30 days of index date	32 (2.0)	14 (0.9)	0.0266
Time to death (days), mean (SD)^b^	22.1 (38.0)	19 (26.7)	0.2887
≥ 1 ED visit or hospitalization	500 (31.7)	392 (24.8)	< 0.0001
≥ 1 ED visit	280 (17.7)	243 (15.4)	0.0765
≥ 1 hospitalization	289 (18.3)	217 (13.8)	0.0006
Death from any cause in those with ≥ 1 hospitalization	29 (10.0)	7 (3.2)	0.0032
Death within 30 days in those with ≥ 1 hospitalization	24 (8.3)	7 (3.2)	0.0083

*COVID-19* coronavirus disease 2019, *ED* emergency department

^a^ Unless otherwise stated

^b^ For patients who died

Cox proportional hazards modeling showed that risk of death after the COVID-19 index date was significantly lower for patients with allergic asthma than nonallergic asthma (hazard ratio, 0.48; 95% CI 0.28–0.83; *P* = 0.0087). However, among the individuals who died, the number of days between index date and death was not statistically significant according to allergic asthma history (Table 2).

### Hospitalization, hospitalization outcomes, and treatment

Patients with nonallergic asthma were more likely to have visited the ED or to have at least 1 hospital admission within 7 days before or 30 days after their index date than patients with allergic asthma. The proportion of patients hospitalized with COVID-19 was greater for patients with nonallergic asthma than allergic asthma (18.3% [*n* = 289] vs 13.8% [*n* = 217]; *P* = 0.0006; Table 3). A similar proportion of those hospitalized in the 2 cohorts were admitted because of their asthma (nonallergic asthma, 73.0% [*n* = 211]; allergic asthma, 75.1% [*n* = 163]). Once patients were hospitalized, there were no significant differences between patients with allergic or nonallergic asthma in terms of need for ICU admission, ventilation, or oxygen therapy support, or in the proportion of patients readmitted (after release) within 30 days.

**Table 3:** Hospitalizations and hospitalization outcomes among patients with a COVID-19 diagnosis (propensity score–matched study cohorts)

Outcome, n (%)	Nonallergic asthma (*n* = 1578)	Allergic asthma (*n* = 1578)	*P* value
Inpatient hospitalization	289 (18.3)	217 (13.8)	0.0006^a^
Asthma hospitalization	211 (73.0)	163 (75.1)	0.5936^a^
Readmission within 30 days	14 (4.8)	8 (3.7)	0.5274^a^
Intensive care unit admission	47 (16.3)	35 (16.1)	0.9677^a^
Noninvasive mechanical ventilation	39 (13.5)	26 (12.0)	0.6146^a^
Invasive mechanical ventilation	31 (10.7)	19 (8.8)	0.4622^a^
Nasal cannula	103 (35.6)	75 (34.6)	0.8016^a^
Extra corporeal membrane oxygenation	0	1 (0.5)	0.4289^b^

*COVID-19* coronavirus disease 2019

^a^ χ^2^ test

^b^ Fisher’s exact test

The proportion of hospitalized patients treated with medications for COVID-19, and the number of medications administered, were similar between the 2 cohorts (Table 4). The exception was dexamethasone, which was administered in 21.2% (*n* = 334) of patients with nonallergic asthma vs 17.4% (*n* = 274; *P* = 0.0068) of patients with allergic asthma, a finding not observed with other glucocorticoids.

**Table 4:** COVID-19 treatments after COVID-19 diagnosis (propensity score–matched study cohorts)

Treatment, n (%)^a^	Nonallergic asthma (*n* = 1578)	Allergic asthma (*n* = 1578)	*P* value^b^
Any	561 (35.6)	509 (32.3)	0.0505^b^
Hydroxychloroquine/chloroquine	59 (3.7)	58 (3.7)	0.9249^b^
Dexamethasone	334 (21.2)	274 (17.4)	0.0068^b^
Methylprednisolone	291 (18.4)	281 (17.8)	0.644^b^
Remdesivir	119 (7.5)	103 (6.5)	0.2654^b^
Tocilizumab	16 (1.0)	13 (0.8)	0.5757^b^
No. of treatments^c^			0.2473
0	1017 (64.5)	1069 (67.7)	
1	354 (22.4)	329 (20.8)	
2	145 (9.2)	135 (8.6)	
3	57 (3.6)	40 (2.5)	
4	5 (0.3)	4 (0.3)	
5	0	1 (0.1)	

*COVID-19* coronavirus disease 2019

^a^ From index date to end of COVID-19–related patient follow-up

^b^ χ^2^ test

^c^ Including those specified above only

### Outpatient visits, and outcomes not requiring hospitalization

The number of outpatient visits for any reason was similar between the cohorts (nonallergic asthma, 48.0% [*n* = 758] vs allergic asthma, 45.9% [*n* = 724]; *P* = 0.2253) The number of outpatient visits for asthma was also similar between patients with nonallergic asthma (36.1% [*n* = 569]) or allergic asthma (38.2% [*n* = 603]; *P* = 0.2253). In addition, COVID-19–related outcomes that did not require hospitalization, including loss or distortion of sense of smell, change in sense of taste, or disturbances of senses of both taste and smell, were reported for less than 2.5% of patients (data not shown), and there were no significant differences between patients with allergic or nonallergic asthma.

### Health care resource use

Patients with nonallergic asthma had a greater rate of hospital resource use (inpatient days and ED visits), a lower rate of outpatient visits for any reason or for asthma, and higher outpatient visits for COVID-19, compared with patients with allergic asthma (Table 5). The overall outpatient resource use rates related to any changes in senses of taste and smell were low, but slightly higher for patients with allergic asthma.

**Table 5:** Hospital and outpatient care resource use after COVID-19 diagnosis (propensity score–matched study cohorts)

Resource counts per 1000 person-days	Nonallergic asthma	Allergic asthma	Rate ratio^a^	95% CI	*P* value^b^
Hospital resource use					
Total inpatient time (days)	18.6	12.5	0.71	0.67–0.75	< 0.001
Emergency department visits	6.7	5.2	0.78	0.72–0.86	< 0.001
Outpatient resource use					
Outpatient visits	40.6	51.4	1.26	1.22–1.31	< 0.001
COVID-19–related outpatient visits	10.3	9.1	0.88	0.82–0.94	0.0004
Asthma-related outpatient visits	9.2	11.7	1.27	1.19–1.37	< 0.001

*COVID-19* coronavirus disease 2019

^a^ Compares allergic asthma with nonallergic asthma

^b^ From χ^2^ test of regression coefficient

## Discussion

Our real-world analysis used a large database (including EHR from health care facilities across most of the United States) to evaluate COVID-19 outcomes in patients with different asthma subtypes. We found that patients with evidence of allergic asthma had a lower risk of hospitalization or death from any cause after diagnosis, compared with patients with nonallergic asthma. However, once hospitalized, the rates of ICU admissions, need for respiratory support, and treatment were similar in both cohorts. In addition, we found that only around 11% of patients with COVID-19 and asthma had allergic asthma, which was a lower proportion than might be expected from epidemiological studies of asthma (at least half and up to 78% of patients with asthma have allergic asthma [22–25]). Although we were unable to estimate the rate of SARS-CoV-2 infection among asthma groups due to the nature of the available data, this may suggest that patients with allergic asthma have a lower risk of SARS-CoV-2 infection than those with nonallergic asthma. Nevertheless, we found that overall, nonallergic asthma was associated with worse outcomes than allergic asthma, which may be important to consider during treatment.

Our findings are in line with previous studies and support that allergic asthma is associated with lower risk of severe COVID-19 outcomes than nonallergic asthma [8, 11, 14]. Our findings also align with a study that found that patients with asthma and rhinosinusitis or allergic rhinitis (i.e., markers of allergic asthma) were less likely to be hospitalized than patients with COVID-19 without these concomitant conditions [26]. More broadly, a number of studies have shown that atopic diseases are associated with milder COVID-19 [27], or less risk of hospitalization due to COVID-19 [28].

The potentially lower risk of SARS-CoV-2 infection in patients with allergic asthma compared with nonallergic asthma could theoretically be explained by differences in expression of the key SARS-CoV-2 cellular receptor, angiotensin-converting enzyme 2 (*ACE2*). *ACE2* expression is upregulated in individuals who are at higher risk of severe COVID-19, such as people with diabetes and obesity, and in those who smoke [29–31]. Jackson et al. evaluated expression of *ACE2* in the airways of in adults and children with asthma and found that *ACE2* expression was lower in the airway of patients with allergic sensitization and asthma compared with patients with nonallergic asthma [29]. Reduced *ACE2* expression combined with expression of immunomodulators could contribute to lower COVID-19 susceptibility for patients with nonallergic asthma, and this hypothesis is supported by a growing body of preclinical and clinical evidence [29, 32–37].

This study had a number of strengths, including a large sample size of patients from across the United States. The data also allowed tracking of patients with COVID-19 from diagnosis through to treatment, and distinguishing of patient symptomology between patients with a positive COVID-19 test and those with a negative COVID-19 test. However, there are a number of limitations that could hinder the applicability and generalizability of the findings, which are mostly related to the database. The major limitation is that ICD-CM-10 codes do not exist for allergic asthma; as a result, the case definitions used for the 2 cohorts were based on current literature, clinical opinion, and available data in the database, which included a positive IgE test, omalizumab use, or a skin prick test ordered by a specialist. Therefore, it is possible some patients may have been misclassified as not having allergic asthma if these data were not available. However, a positive skin prick test is thought to be likely if it has been ordered by a specialist, and omalizumab is indicated for use in allergic asthma and not in nonallergic asthma. Although it is possible that omalizumab could be prescribed for chronic spontaneous urticaria in patients with nonallergic asthma, and these patients could have been misclassified as having allergic asthma, the presence of asthma as a comorbid condition in patients with chronic spontaneous urticaria is low [38]. In addition, the database does not cover all health care facilities in the United States and may not be nationally representative of patients who attend smaller ambulatory practices. The outcomes assessed in this study are also limited because causes of death were not available in the database and the analysis of deaths could only be reported as deaths due to any cause and not specifically attributed to COVID-19. Finally, our understanding of COVID-19 is dynamic and rapidly advancing, and the current analysis therefore may not include any newly identified risks or other factors, such as the impact of treatments, vaccines, or new variants.

## Conclusions

In summary, this real-world retrospective analysis of a large EHR database from the United States found that allergic asthma was associated with a lower risk of poor outcomes, including a lower risk of death, from COVID-19 compared with nonallergic asthma. Among patients with asthma and SARS-CoV-2 infection, a lower-than-expected proportion had allergic asthma, which may suggest a lower infection rate. These findings contribute to the growing body of evidence on SARS-CoV-2 infections in patients with asthma, including differences in outcomes for asthma subtypes.

## Data Availability

The data that support the findings of this study are available from Optum Inc, but restrictions apply to the availability of these data. For this study, the data were used under license and so are not publicly available. Data are available from the authors upon reasonable request and with permission from Optum Inc.

## Abbreviations

ACE2: Angiotensin-converting enzyme 2
CDC: Centers for Disease Control and Prevention
COVID-19: Coronavirus disease 2019
ED: Emergency department
EHR: Electronic health record
ICD-10-CM: *International Classification of Diseases, Tenth Revision, Clinical Modification*
ICU: Intensive care unit
IgE: Immunoglobulin E
SARS-CoV-2: Severe acute respiratory syndrome coronavirus 2
